# A new CBCT evaluation for individual assessment of midpalatal suture maturation: a retrospective analysis

**DOI:** 10.3389/froh.2025.1630883

**Published:** 2026-01-16

**Authors:** Andrea Boggio, Gianluigi Fiorillo, Enrico Razzani, Beatrice Manes Gravina, Gualtiero Mandelli, Raffaele Vinci, Fabio Castellana, Giorgio Gastaldi

**Affiliations:** 1 Department of Dentistry, Vita-Salute San Raffaele University, Milan, Italy; 2 Department of Interdisciplinary Medicine (DIM), University of Bari Aldo Moro, Bari, Italy; 3 Interateneo Department of Physics, University of Bari, Bari, Italy

**Keywords:** Angelieri's classification, assessment, cone-beam computed tomography (CBCT), coronal slices evaluation, diagnosis, maxillary expansion, midpalatal suture maturation

## Abstract

**Introduction:**

The maturation of the midpalatal suture is a critical factor in determining the most appropriate maxillary expansion technique. Angelieri et al. introduced a CBCT-based staging system that shifted the focus from chronological age to individual anatomical assessment. However, inter-examiner variability and challenges in evaluating intermediate stages (C and D) have raised concerns about the diagnostic reliability of axial-only CBCT analysis. This study investigates whether the addition of standardized coronal CBCT sections to traditional axial assessment can improve diagnostic precision - particularly in borderline cases - by revealing morphological variations that may not be evident in axial views alone.

**Materials and methods:**

34 CBCT scans were retrospectively analyzed. Each midpalatal suture was assessed using both the conventional axial plane method proposed by Angelieri and a coronal view evaluation performed on three standardized slices (anterior, middle, posterior). The study focused on evaluating concordance between the two modalities, identifying regional discrepancies and analyzing ossification patterns, particularly in intermediate stages.

**Results:**

Full concordance between axial and coronal assessments was observed in 23 out of 34 cases, supporting the overall consistency of the axial view method. However, discrepancies emerged primarily in stage C, where 8 of the 11 discordant cases were concentrated. In most of these cases, at least one coronal slice revealed a more advanced ossification stage than suggested by axial analysis. Additionally, atypical anterior-to-posterior ossification patterns were documented in a minority of cases.

**Discussion:**

While our findings do not question the general validity of Angelieri's staging, they suggest that an exclusive reliance on axial views may, in some cases, underestimate the degree of suture maturation. The integration of coronal slices can improve diagnostic resolution in transitional stages, offering a more specific view of the suture's complexity. This multimodal approach may help reduce interpretive subjectivity and potentially limit inter-examiner variability.

## 1 Introduction

Skeletal maxillary constriction is a common condition affecting the craniofacial region and frequently encountered in orthodontics (1).

This malocclusion is associated with several etiologic factors, most notably prolonged childhood habits such as oral breathing, atypical swallowing, and persistent thumb or pacifier sucking. These dysfunctional habits disrupt normal tongue posture, lead to lip incompetence, and alter the balance of the perioral musculature, ultimately impeding proper development and physiological expansion of the maxillary arch (2–5).

Transverse deficiencies of the maxilla are traditionally treated through palatal expansion techniques, including Rapid Maxillary Expansion (RME), Slow Maxillary Expansion (SME), Surgically Assisted Rapid Maxillary Expansion (SARME), and Miniscrew-Assisted Rapid Palatal Expansion (MARPE). These procedures aim to separate the midpalatal suture by exerting orthopedic forces that induce collagen fiber elongation, bone resorption, and subsequent new bone formation to stabilize the gained expansion (6). The midpalatal suture undergoes progressive changes during growth. It typically begins obliteration between ages 15 and 18 and may be completely fused between 25 and 35 years of age. However, this ossification process varies considerably among individuals, making chronological age an unreliable indicator for determining suture maturity (7–9).

Suture ossification increases the resistance to expansion, thus requiring different intensities and modes of force applied in maxillary expansion techniques.

Applying an inappropriate expansion protocol without considering the suture's maturity can result in numerous adverse effects, including: Periodontal complications (10, 11), Buccal tipping of anchorage elements (3, 12–14), Bone injuries (12), Pain (13), Limited dentoalveolar rather than skeletal expansion (3, 7, 15), Root reabsorption (3, 10), Mucosal ulcers (3, 15, 16).

Traditionally, the therapeutic choice for palatal expansion was mainly based on the chronological age of the patient, assuming that it corresponded to a specific stage of suture maturation, thus differentiating different expansion techniques between preadolescents, adolescents, young adults, and adults (3). However, recent studies show that chronological age is not a reliable parameter for assessing the maturation status of the midpalatine suture. In addition, high individual variability in the developmental stages of median palatine suture fusion has also been observed in relation to gender (7, 17–19). Furthermore, there is no consensus in the literature regarding the indicative age for SARME (20–22).

Various attempts to assess suture maturation—including histological evaluations, occlusal radiography, and animal studies using computed tomography—have proven inconsistent and diagnostically limited (22–27).

To address the absence of reliable clinical indicators, Angelieri et al. introduced a method in 2013 for assessing midpalatal suture maturity using cone-beam computed tomography (CBCT). This method evaluates the suture in axial sections and classifies its morphology into five distinct stages (A–E) (7, 15), representing one of the main diagnostic references nowadays.

Although biopsy is the current gold standard for assessing midpalatal suture (MPS) maturation, it is unfeasible to perform in patients. Conversely, serial occlusal radiographs are limited in diagnostic quality due to the superimposition of adjacent anatomical structures (7, 28). A non-invasive imaging methods such as CBCT allows three-dimensional rendering of the maxillofacial complex without overlapping structures and delivers a lower radiation dose to patients compared with conventional medical CT.

Nevertheless, despite its popularity, this diagnostic approach has been criticized for its subjective interpretation, raising concerns about interobserver reliability (28, 29).

Despite its widespread adoption, the reliability of this diagnostic criterion is often debated due to its intrinsic subjectivity (28, 29). Moreover, a significant individual variability in treatment response is still observed, highlighting the potential value of integrating additional diagnostic methods to enhance the precision and reliability of suture maturation assessment.

Therefore, this study aims to enhance Angelieri's axial view-based classification by incorporating observations from three additional coronal CBCT slices: posterior, central, and anterior. By broadening the dimensional analysis of the suture, this integration seeks to offer clinicians a more detailed and objective framework for evaluating ossification status and selecting the most appropriate expansion strategy.

This protocol aims to:

- Verify the concordance between maturation stages observed in the axial plane and those identified in coronal slices, evaluating the entire thickness of the suture.
- Explore the posterior-to-anterior ossification pattern, the typical progression described in the literature.
- Identify potential diagnostic discrepancies between the two observation modalities and analyzing whether these discrepancies are due to increased or reduced ossification.

## 2 Materials and methods

This retrospective observational study was conducted at the San Raffaele Hospital, Department of Dentistry, between 2019 and 2024. It adhered to the principles of the Declaration of Helsinki and received approval from the Ethics Committee of Vita-Salute San Raffaele University (approval code DIG-RETRO-1/2021).

### 2.1 Patient selection

CBCT scans acquired for clinical diagnostic purposes were retrospectively evaluated. Inclusion criteria were: patients aged ≥5 years of any gender, good general health, presented with a transverse maxillary deficiency, signed informed consent. Exclusion criteria were: presence of systemic diseases, craniofacial anomalies or syndromes, previous orthodontic treatment, and low-quality CBCT scans.

For analytical purposes, subjects were stratified into four age groups based on developmental stages commonly referenced in the literature: Group 1 (5 to <11 years), Group 2 (11 to <14 years), Group 3 (14–18 years), and Group 4 (>18 years). This categorization aimed to assess the distribution of midpalatal suture maturation stages in relation to age and evaluate potential trends associated with craniofacial growth. The selected age intervals reflect key transitional periods in skeletal and dental development, facilitating a more precise interpretation of maturation patterns across different developmental stages.

### 2.2 CBCT acquisition and standardization

CBCT scans were performed using the Sirona Orthophos XG-3D system under ALARA principles, with a mini field of view (8 × 8 cm), scan time between 8.9 and 14 s, and a resolution of 0.1–0.2 mm. During acquisition, patients were positioned with the Frankfurt horizontal plane parallel to the ground. CBCT acquisition was performed by the same two operators each time.

Scans were analyzed using Real-Guide® software.

To ensure the consistency and accuracy of the CBCT analysis, each case underwent a procedure to standardize the orientation of the skull before the analysis:

- In the axial view, image orientation was standardized by aligning the anterior and posterior nasal spines along the median plane.
- In the coronal view, the vertical reference line of the software was centered on the midsagittal plane of the skull.
- In the sagittal view, the skull was oriented to ensure that the postero-anterior axis of the palate was as parallel as possible to the software's horizontal reference line.

### 2.3 Assessment of midpalatal suture maturation

**Figure 1:**
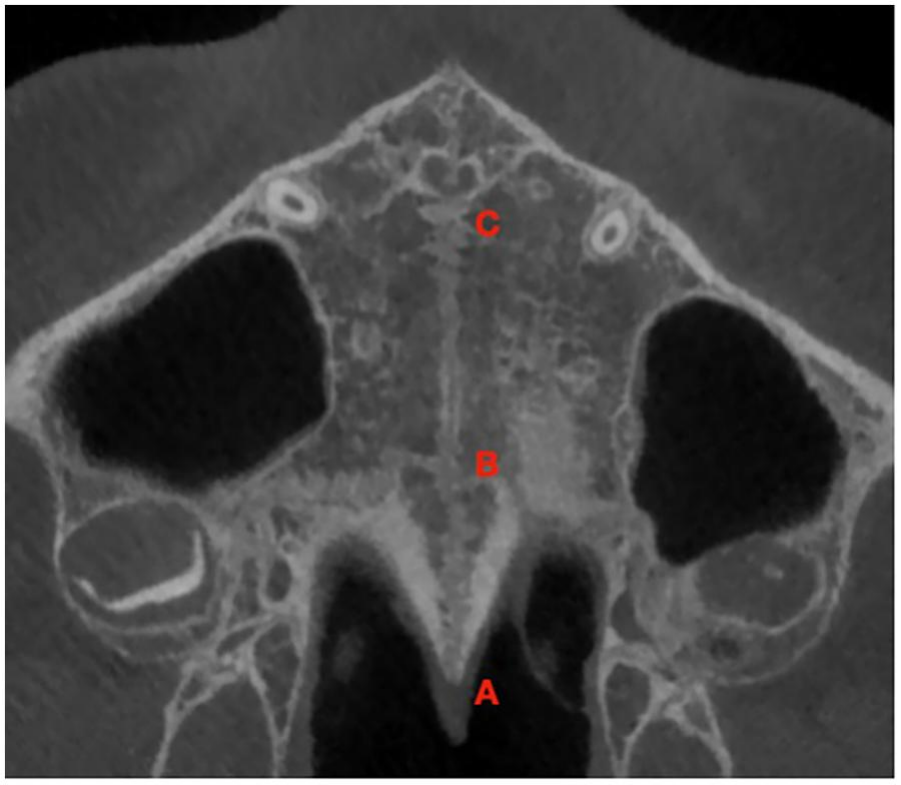
Identification of the anatomical reference points along the palatal midline used for standardized coronal slice positioning. Point A: posterior edge of the palatine bone; point B: transverse palatine suture; point C: posterior border of the incisive foramen. Points were identified in the sagittal view and referenced along the palatal midline.

The median palatine suture was assessed on standardized axial slices (Figure 1) and classified into one of five maturation stages (A–E) according to Angelieri et al. (7) by two experienced examiners. In anatomically complex cases (curved and thick palates), two central axial slices (anterior and posterior) were analyzed as proposed by Angelieri et al. (7, 15).Final axial classifications were established by consensus following joint evaluation of each case.

### 2.4 Coronal slice acquisition

**Figure 2:**
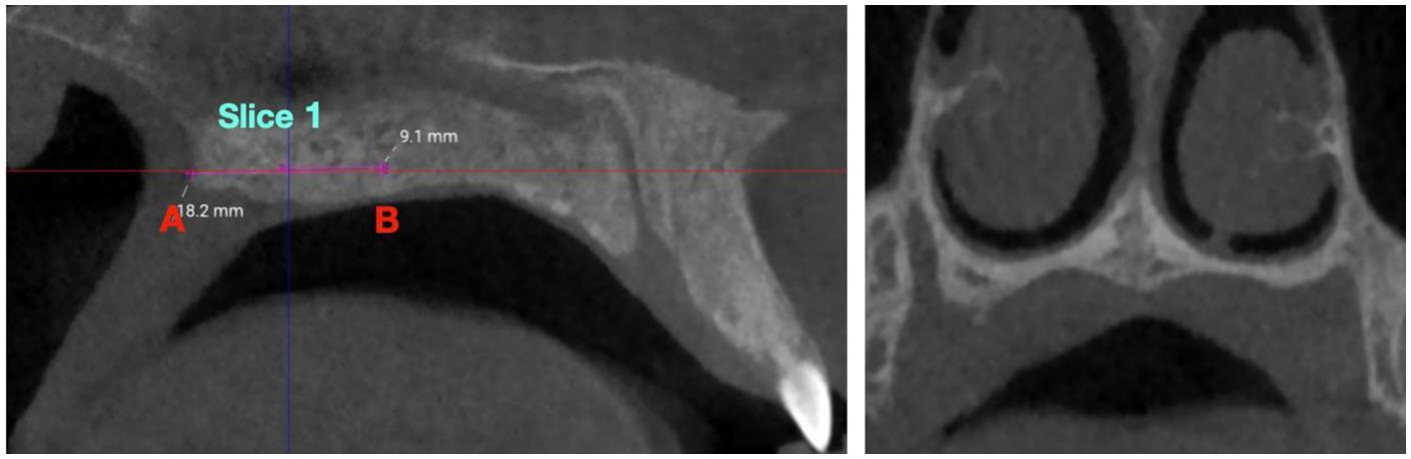
Coronal slice 1 (posterior) location. The slice was obtained at the midpoint between point A (posterior edge of the palatine bone) and point B (transverse palatine suture), along the palatal midline.

**Figure 3:**
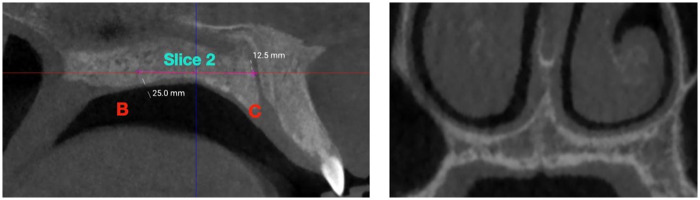
Coronal slice 2 (central) location. The slice was obtained at the midpoint between point B (transverse palatine suture) and point C (posterior border of the incisive foramen), along the palatal midline.

**Figure 4:**
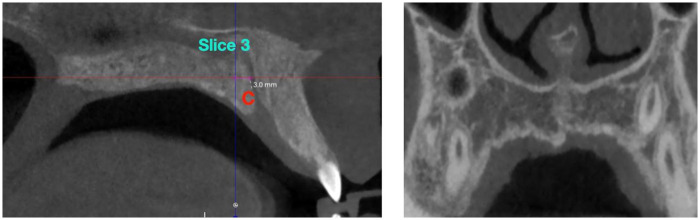
Coronal slice 3 (anterior) location. The slice was obtained 3 mm posterior to point C (posterior border of the incisive foramen), along the palatal midline.

Three coronal slices were obtained per subject at standardized locations (Figure 1):

- Slice 1 (Posterior): midway between the posterior edge of the palatine bone (point A) and the transverse palatine suture (point B). (Figure 2)
- Slice 2 (Central): midway between point B and the posterior border of the incisive foramen (point C). (Figure 3)
- Slice 3 (Anterior): 3 mm posterior to point C. (Figure 4)
  All points were identified in the sagittal view and referenced along the palatal midline.

### 2.5 Data collection and analysis

Data were tabulated in Microsoft Excel. For each subject, the midpalatal suture observed in the axial slice was compared with the view of the same suture in the three coronal slices (S1, S2 and S3).

CBCT scans were evaluated by two experienced examiners following a standardized image orientation and evaluation protocol. Assessments were conducted jointly, and final classifications regarding axial staging and coronal concordance were reached by consensus after discussion of each case.

The collected data were systematically organized into four tables, each designed to highlight a specific aspect of the concordance between axial and coronal observations:

- General Concordance Table: This table lists, for each subject, the reference maturation stage of the midpalatal suture based on Angelieri's axial classification (Stages A to E), and indicates the number of coronal slices (0, 1, 2, or 3) that exhibited concordance with the axial stage.
- Single Concordance Table: For cases in which only one coronal slice matched the axial classification, this table identifies which specific slice (S1, S2, or S3) demonstrated concordance.
- Moderate Concordance Table: In cases where two out of three coronal slices matched the axial stage, this table specifies which pair of slices showed concordance.
- Table of increased or reduced ossification: Focused on the moderate concordance group, this table evaluates whether the non-concordant coronal slice exhibited a relative increased or reduced ossification compared to the axial reference stage. This assessment was limited to moderate concordance cases, as only one case of single concordance was identified and is reported separately.

### 2.6 Statistical analysis

The entire sample was divided into five groups according to Agelieri's classification. The normal distributions of the quantitative variables were tested using the Kolmogorov–Smirnov test. Therefore, data were reported as mean ± standard deviation (M ± SD), median (iqr) for continuous measures and frequency and percentages (%) for all categorical variables. A nonparametric approach was used to assess statistical differences between groups. The Kruscal Wallis sum rank test for independent samples was adopted to evaluate any statistical differences between groups for continuous variables and the Chi square test or Fischer exact test for categorical ones. In order to assess any statistically significant differences between age groups in the distribution by sex, a continence table was constructed using Fisher's exact test. In order to assess the concordance between the Angelieri classification and the Coronal Slices evaluated, a continence table was constructed using Fisher's statistical test to evedicate any statistically significant differences (Supplementary Table S1). In order to highlight statistically significant differences in the distribution of the Angelieri's classification with respect to sex in different age groups, a continence table was constructed using Fisher's exact test. In order to show statistically significant differences in the distribution of concordance between the Angelieri's classes and the evaluated Coronal Slices, two continence tables were constructed respectively to emphasize in which coronal cutoff there was concordance with the Angelieri classification and to highlight this relationship only in the group of “Moderate” concordance. In order to assess any statistically significant difference in direction of difference between Coronal Slices and Angelieri's classification, a contingence table using fisher's exact test was adopted.

## 3 Results

A total of 44 baseline CBCT scans acquired for clinical purposes were initially considered for inclusion. Following quality assessment, 10 scans were excluded due to insufficient image quality (e.g., motion artifacts, blurring). The final study sample comprised 34 subjects (25 females and 9 males), aged between 9.2 and 52.1 years, all with no history of prior orthodontic treatment. Table 1 shows sex distribution between age groups.

**Table 1:** Sex distribution according to age groups.

Description parameters	Age groups				
	5 to <11	11 to <14	14–18 years	>18 years	*p*
Female	2 (50.00)	12 (85.70)	5 (83.30)	6 (60.00)	0.28
Male	2 (50.00)	2 (14.30)	1 (16.70)	4 (40.00)	

All data are categorical and shown as *n* (%).

Fischer's exact test was used.

**Figure 5:**
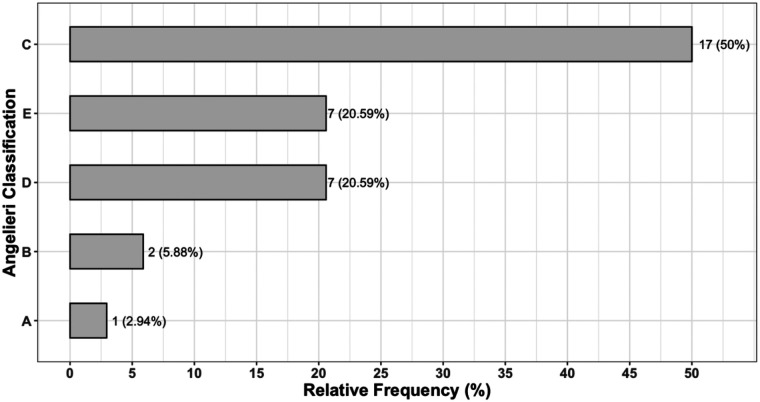
Relative frequency plot of Angelieri classification.

The distribution of midpalatal suture maturation stages, classified according to Angelieri et al. (7), is presented in Figure 5. Stage A was observed in 1 subject (2.94%), stage B in 2 subjects (5.88%), stage C in 17 subjects (50%), stage D in 7 subjects (20.59%), and stage E in 7 subjects (20.59%).

Further stratification of the data was performed to evaluate the relationship between suture maturation stages, age, and sex. Tables 2, 3 report the frequency of each maturation stage across the age groups, with sex-specific distributions detailed accordingly.

**Table 2:** Description of the whole sample according to Angelieri classification.

Description parameters	Group A		Group B		Group C		Group D		Group E		
	mean ± sd	median (iqr)	mean ± sd	median (iqr)	mean ± sd	median (iqr)	mean ± sd	median (iqr)	mean ± sd	median (iqr)	*p* *
Proportions [n (%)]	1 (2.90)		2 (5.90)		17 (50.00)		7 (20.60)		7 (20.6)		
Concordance (n)	3 ± NA	3 (0)	2.5 ± 0.707	2.5 (0.5)	2.471 ± 0.624	3 (1)	2.714 ± 0.488	3 (0.5)	3 ± 0	3 (0)	0.22
Age (years)	9.20 ± NA	9.20 (0)	20.65 ± 15.486	20.65 (10.95)	13.472 ± 2.748	13 (1.8)	16.929 ± 6.32	14 (6.65)	31.917 ± 16.989	25.1 (30.1)	**<0**.**01**
Age group											
5 to <11 years	1 (100.00)		1 (50.00)		2 (11.80)		–		–		**<0**.**01**
11 to <14 years	–		–		11 (64.70)		3 (42.90)		–		
14–18 years	–		–		2 (11.80)		2 (28.60)		2 (28.60)		
>18 years	–		1 (50.00)		2 (11.80)		2 (28.60)		5 (71.40)		
Sex											
Female	–		1 (50.00)		13 (76.50)		5 (71.40)		6 (85.70)		0.45
Male	1 (100.00)		1 (50.00)		4 (23.50)		2 (28.60)		1 (14.30)		

All data are shown as mean ± sd, median (iqr) for continuous variables and as *n* (%) for categorical ones.

* Wilcoxon 'sum rank test for continuous variables and Fisher's exact test for categorical ones.

**Table 3:** Contingence table of sex and Angelieri classification according to age group.

Angelieri's stage	5 to <11 yo		
	F	M	*p* ^*^
A	0 (0)	1 (2.94)	0.13
B	0 (0)	1 (2.94)	
C	2 (5.88)	0 (0)	
D	0 (0)	0 (0)	
E	0 (0)	0 (0)	
	11 to <14 yo		
	F	M	
A	0 (0)	0 (0)	0.99
B	0 (0)	0 (0)	
C	9 (26.47)	2 (5.88)	
D	3 (8.82)	0 (0)	
E	0 (0)	0 (0)	
	14–18 yo		
	F	M	
A	0 (0)	0 (0)	0.30
B	0 (0)	0 (0)	
C	2 (5.88)	0 (0)	
D	1 (2.94)	1 (2.94)	
E	2 (5.88)	0 (0)	
	>18 yo		
	F	M	
A	0 (0)	0 (0)	0.20
B	1 (2.94)	0 (0)	
C	0 (0)	2 (5.88)	
D	1 (2.94)	1 (2.94)	
E	4 (11.76)	1 (2.94)	

* Fisher's exact test.

### 3.1 Coronal view agreement with angelieri staging

Table 4 shows the level of concordance between the axial morphological classification of the midpalatal suture (based on Angelieri's staging) and the corresponding evaluations in the three standardized coronal slices. The results are as follows:

- Stage A: The only case showed full concordance across all three coronal slices.
- Stage B: Of the two cases, one exhibited complete concordance, while the other showed moderate concordance (agreement in two slices).
- Stage C: Among 17 cases, 1 showed partial concordance (agreement in one coronal slice), 7 showed moderate concordance, and 9 demonstrated complete concordance.
- Stage D: Out of 7 cases, 5 exhibited full concordance, while 2 showed moderate concordance.
- Stage E: All 7 cases displayed complete concordance with axial staging in all three coronal slices.

**Table 4:** Contingence table of coronal slices (CS) concordance with Angelieri's classification. All data are categorical variables.

Angelieri's stage	0 CS	1 CS	2 CS	ALL CS	*p*
A	–	–	–	1 (4.30)	0.35
B	–	–	1 (10.00)	1 (4.30)	
C	–	1 (100.00)	7 (70.00)	9 (39.10)	
D	–	–	2 (20.00)	5 (21.70)	
E	–	–	–	7 (30.40)	

Fisher's exact test was used.

The following table (Table 5) aims to specify in which of the three coronal slices (S1, S2 or S3) there was concordance with Angelieri's stage, exclusively for cases of partial concordance (only one coronal slice in agreement with Angelieri's stage).

**Table 5:** Specifies in which one of the three coronal slices (S1, S2, or S3) there was concordance with Angelieri's stage.

Angelieri's stage	S1	S2	S3	*p*
A	0	0	0	0.99
B	0	0	0	
C	0	0	1 (100.00)	
D	0	0	0	
E	0	0	0	

Fisher's exact test was used.

The single case of a midpalatal suture with partial concordance showed agreement with Angelieri's Stage C only in Coronal Slice 3. In Coronal Slices 2 and 3, very advanced ossification was observed, rendering the midpalatal suture indistinguishable.

The following table (Table 6) aims to specify which two coronal slices were in agreement with Angelieri's stage, exclusively for cases of Moderate Concordance (two coronal slices out of three in agreement with Angelieri's stage).

- Stage A: No cases of midpalatal suture at stage A were found to be discordant.
- Stage B: The only case of stage B showing concordance in 2 slices exhibited agreement with Angelieri's stage in slices 2 and 3.
- Stage C: Two cases showed concordance with Angelieri's stage in slices 1 and 2. Two cases showed concordance in slices 1 and 3. Three cases showed concordance in slices 2 and 3.
- Stage D: One case showed concordance with Angelieri's stage in slices 1 and 3. One case showed concordance in slices 2 and 3.
- Stage E: No cases of midpalatal suture at stage E were found to be discordant in the coronal slice evaluation.

**Table 6:** Specifies which two coronal sections were in agreement with Angelieri's stage, exclusively for cases of Moderate Concordance.

Angelieri's stage	S1–S2	S1–S3	S2–S3	*p*
A	0	0	0	0.99
B	0	0	1 (20.00)	
C	2 (100.00)	2 (66.70)	3 (60.00)	
D	0	1 (33.30)	1 (20.00)	
E	0	0	0	

Fisher's exact test was used.

Table 7 presents the nature of discordance in cases of moderate concordance, specifying whether the mismatched coronal slice (S1, S2, or S3) exhibited increased or reduced ossification of the midpalatal suture compared to the axial reference stage based on Angelieri et al.method.

- S1: Midpalatal suture in slice 1 was found with increased ossification in one case of stage B (axial reference stage) and in 3 cases of stage C, while with reduced ossification in one case of reference stage D.
- S2: Midpalatal suture in slice 2 was characterized by increased ossification in two cases of stage C and in one case of stage D.
- S3: Midpalatal suture in slice 3 was found with increased ossification in two cases of stage C.

**Table 7:** Description of the ossification seen in the discordant coronal slice relative to the axial reference stage.

Angelieri's stage	S1		
	Increased ossification	Reduced ossification	*p*
A	0 (0.00)	0 (0.00)	0.40
B	1 (25.00)	0 (0.00)	
C	3 (75.00)	0 (0.00)	
D	0 (0.00)	1 (100.00)	
E	0 (0.00)	0 (0.00)	
	S2		
	Increased	Reduced	*p*
A	0 (0.00)	0 (0.00)	0.40
B	0 (0.00)	0 (0.00)	
C	2 (66.70)	0 (0.00)	
D	1 (33.30)	0 (0.00)	
E	0 (0.00)	0 (0.00)	
	S3		
	Increased	Reduced	*p*
A	0 (0.00)	0 (0.00)	0.99
B	0 (0.00)	0 (0.00)	
C	2 (100.00)	0 (0.00)	
D	0 (0.00)	0 (0.00)	
E	0 (0.00)	0 (0.00)	

Fisher's exact test was used.

## 4 Discussion

### 4.1 Axial assessment compared to coronal slices analysis

Recent literature has highlighted that chronological age is an insufficient parameter to accurately predict the maturation phase of the midpalatal suture, and consequently, to guide orthodontic treatment planning **(7, 23)**.

Histological studies by Persson and Thilander (23) documented fusion of the midpalatal suture in the posterior palate in young individuals (15 years for a female, 21 years for a male), while in older patients (aged 27, 32, 54, and even 71 years) no fusion was observed. These histological findings are in contrast with traditional literature – for example the studies of Hass (30) and Melsen (31) -, which suggests limited effectiveness of rapid maxillary expansion (RME) in patients over 25 years of age.

In view of the poor predictability of chronological age, Revelo and Fishman (27) introduced the individual assessment of the midpalatal suture using occlusal radiographs. However, Wehrbein and Yildizhan (25) subsequently demonstrated, through histological analysis, that occlusal radiographs are unreliable for diagnosing suture fusion due to the overlap of the vomer and other anatomical structures in the palatal region.

In 2013, Angelieri et al. (7) introduced CBCT as an innovative diagnostic approach to examine midpalatal suture maturation, noting significant variability in the distribution of maturation stages across different age groups. In fact, they found that all stages of ossification could occur as early as age 11. Thus, chronological age appears increasingly irrelevant for both diagnosis and therapeutic decision-making in orthodontics.

Therefore, Angelieri emphasizes the importance of radiographic analysis of the suture for each individual case, specifically through CBCT evaluation.

However, previous studies have highlighted significant inter-examiner variability in assigning maturation stages, particularly for sutures in transitional phases between two stages. This diagnostic variability can lead to discrepancies in clinical decisions and treatment selection, thus compromising the standardization and reproducibility of the method (32).

Both Angelieri's original classification and the present integrative approach are inevitably influenced by interindividual variability. Such variability arises from several concurrent factors: 1) inherent anatomical differences among patients (29) - such as variations in sutural morphology, bone density, and the degree of interdigitation - which can alter the radiographic appearance of the suture; 2) the subjective component involved in interpreting CBCT greyscale transitions, particularly in intermediate maturation stages where partial ossification may mimic adjacent structures (32); and 3) the operator's experience, which strongly affects the ability to orient images correctly, identify the sutural path, and discern subtle morphological cues (28, 32). These factors, compounded by potential image artifacts or differences in voxel size and contrast resolution across CBCT devices, contribute to diagnostic heterogeneity even when standardized criteria are applied. Recognizing these sources of variability reinforces the rationale for integrating additional coronal slices into the assessment protocol, as multi-planar visualization can mitigate interpretive subjectivity and improve diagnostic consistency, especially in borderline cases.

Additional factors contribute to these challenges: the quality of radiographic images (which can affect the visibility of anatomical details), the requirement for advanced expertise in using diagnostic software and the specific experience of the examiner. Errors in the orientation of axial images or in the interpretation of suture characteristics may further amplify the risk of inaccurate diagnoses.

The present study proposes an integrative diagnostic approach, combining the analysis of the suture in the axial plane, as described by Angelieri, with the observation of three standardized coronal slices.

Tables 2, 3 - structured following the model presented by Angelieri et al. (7)— illustrate the distribution of midpalatal suture maturation stages in our study sample. The low frequency of stages A and B may be attributed to the fact that these maturation stages are typically observed at very early ages, where there is rarely a diagnostic justification for performing a CBCT.

The data highlight a significant variability in maturation stages, irrespective of the patient's age. These findings support the notion that while chronological age may be useful, it cannot be considered a reliable indicator of the midpalatal suture's ossification stage (7, 23).

Other variables have been extensively reported in the literature as contributing factors to the progression of ossification and interdigitation of the midpalatal suture. Beyond the now weak criterion of chronological age, two critical factors are the patient's sex and the resistance to expansion exerted by circummaxillary sutures.

Numerous studies show that, on average, ossification of the midpalatal suture occurs earlier in females than in males (26, 30, 33). However, Angelieri (7), Persson and Thilander (23) illustrated a significant variability in the distribution of maturation stages of the midpalatal suture across both sexes and age groups. Thus, while sex and age offer valuable insights, they cannot be considered as standalone indicators for determining a patient's diagnostic profile or guiding therapeutic options.

In our study sample, within the childhood and adolescent age groups, a higher prevalence of females was observed in stages C (2 females against 0 males in the 5 to <11yo range and 9 females against 2 males in the 11-<14yo range) and D (3 females against 0 males in the 11 to <14yo range) of midpalatal suture maturation. This finding is in agreement with the literature, which suggests that the ossification of the suture generally occurs earlier in females than in males. However, it should be emphasized that the predominance of females in our sample might introduce a bias, limiting the reliability of this result as a definitive indicator.

Given the limited number of cases in stages A and B within the study sample, and considering the complete agreement between axial and coronal evaluations for stage E, this section will examine only discrepancies and concordances (between axial and coronal slices) concerning stages C and D. Focusing on these two stages is particularly crucial for clinicians, as they often represent critical situations, describing a suture in the midst of interdigitation and ossification. The analysis of the 34 cases included in this study has provided significant insights into the maturation of the midpalatal suture, contributing to a deeper understanding of its ossification process and diagnostic challenges.

Complete concordance between the three coronal slices and Angelieri's axial staging was observed in 23 out of 34 cases. This high proportion supports the general diagnostic validity of the axial method, particularly for well-defined maturation stages such as A, B, and E. The findings from our sample do not provide sufficient statistical evidence to question the diagnostic validity of Angelieri's classification. While our results show a generally good level of concordance between the two methods, they also highlight some variability—especially in intermediate stages—that may suggest potential areas where additional imaging perspectives could be beneficial.

Stage C emerged as the most diagnostically complex stage, with 7 of the 10 moderate concordance cases belonging to this group. The dynamic and morphologically heterogeneous nature of this stage may explain the observed discrepancies, as rapid ossification processes can result in variable appearances depending on the section plane and precise region analyzed.

In the 10 cases of moderate concordance (2 out of 3 coronal slices in agreement with axial classification), the discordant coronal slice predominantly involved signs of increased ossification compared to the axial interpretation (Table 7). Specifically:

- In slice 1 (posterior slice), four cases showed increased ossification of the midpalatal suture, while one showed a reduced ossification (relative to the axial reference stage).
- In slice 2 (central slice), the three discordant cases displayed excess ossification;
- For both cases in which the discordant cut was slice 3 (anterior slice), the midpalatal suture was found with increased ossification compared to the axial reference stage.

This pattern suggests a potential risk of underestimating the maturation stage when relying exclusively on axial imaging.

Interestingly, 4 cases displayed greater ossification anteriorly rather than posteriorly, as an inversion of the expected posterior-to-anterior maturation gradient typically described in literature. Although preliminary, this observation invites further exploration into potential anatomical or biological factors that may influence ossification directionality.

Table 4 presents the contingency distribution between Angelieri stages and the number of concordant coronal slices. Fisher's exact test was applied to evaluate the association between axial staging and coronal evaluation. While Stage C is the most populated category, it also shows the highest variability in coronal slice agreement, with 8 out of 17 cases showing discrepancies. Despite this, the *p*-value (0.35) does not reach statistical significance, indicating that the discrepancies observed are not sufficient to undermine Angelieri's method's validity. Nonetheless, the existence of these discordant cases strengthens the rationale for complementary diagnostic methods, such as the coronal slices evaluation, particularly in borderline or transitional stages.

The frequent observation of increased ossification in discordant cases implies that the axial views alone may underestimate the suture's maturation stage, especially in stages C and D. Coronal slices could provide additional context, particularly useful when axial assessment appears ambiguous or borderline

### 4.2 Limitations of the research

This study presents certain limitations that may impact the generalizability of its findings. Firstly, the sample comprises 34 CBCT scans, carefully selected following stringent inclusion and exclusion criteria. Expanding future studies with larger sample sizes may further validate these findings and facilitate the broader applicability of results to the general population, ultimately enhancing their clinical relevance.

Another limitation concerns the small number of midpalatal sutures observed in stages A and B, which are more commonly found in younger age groups. However, CBCT imaging is not frequently indicated for such young patients due to diagnostic justification guidelines. Although this limited representation of earlier stages could influence comparisons among different maturity levels, it aligns with current clinical indications and radiation exposure guidelines.

Additionally, the study used only three coronal sections for analytical purposes. However, during the actual analysis process, the entire extent of the suture was evaluated through coronal views, which were then subdivided into three representative areas to aid comprehension. Nonetheless, as with Angelieri's approach in assessing the entire thickness of the suture from the axial view (7, 15, 34), clinicians are advised not to restrict their evaluation to only three coronal sections but rather to examine the entire sutural morphology in the CBCT's coronal perspective.

Another factor worth considering is the role of the circummaxillary sutures, which may significantly contribute to the observed variability. The mechanical resistance generated by these structures during maxillary expansion can influence both the ossification pattern of the midpalatal suture and the overall response to treatment. Previous studies, such as that by Garib et al. (35), have highlighted the importance of circummaxillary sutures as resistive forces during maxillary expansion, noting their potential impact on asymmetric outcomes and variations in the effectiveness of the expansion process.

In this study, the potential influence of circummaxillary sutures was not directly evaluated. Future research integrating the analysis of these structures into the assessment of midpalatal suture maturation could provide a deeper understanding of the variability observed and further refine diagnostic and therapeutic protocols.

A limitation of the present study is the absence of a formal assessment of inter- and intra-rater reliability. Although CBCT scans were evaluated by two experienced examiners following a strict and standardized image orientation and evaluation protocol, and final classifications were reached by consensus, no independent or repeated assessments were performed to quantitatively measure observer agreement. Consequently, the reproducibility of the proposed axial–coronal evaluation method across different observers cannot be fully established. Future studies should incorporate independent assessments and formal reliability analyses to further validate the proposed coronal assessment approach.

### 4.3 Towards personalized protocols

Adopting a combined axial and coronal evaluation of the midpalatal suture can help in developing more tailored treatment strategies, reducing the risk of complications associated with incomplete diagnostic approaches.

Isfeld et al. (9) underscored the limitations of existing methods for assessing mid-palatal suture maturation, highlighting that no single imaging technique or classification system, including CBCT-based staging, has been validated against histological standards. This lack of validation supports the need for clinicians to adopt a multifaceted diagnostic approach, combining various imaging perspectives and criteria to improve accuracy. Given the diagnostic challenges of intermediate stages, our findings reinforce the value of integrating axial and coronal CBCT views to provide a more comprehensive assessment of suture maturation.

Chatwani et al. (32) highlighted substantial inter-examiner variability, even among experienced orthodontists, in classifying suture maturation stages according to Angelieri's method (7), pointing to the significant role of examiner experience and the inherent subjectivity in this diagnostic process. This variability, particularly pronounced in cases with borderline or intermediate features, suggests that single-view assessments may risk overlooking subtle diagnostic nuances.

Incorporating multiple CBCT perspectives, specifically both axial and coronal views, could mitigate the subjectivity inherent to single-view evaluations, offering a more robust and comprehensive visualization of suture morphology.

In conclusion, the present study underscores several clinically relevant insights:

- The combined use of axial and coronal CBCT evaluations could enhance the reliability of midpalatal suture staging, particularly in the diagnostically challenging intermediate stages C and D.
- The observed discrepancies—mostly characterized by more advanced ossification in coronal sections compared to axial views—suggest that relying exclusively on axial assessment may in some cases lead to an underestimation of suture maturation.
- The identification of variable ossification patterns, including atypical anterior-to-posterior progression, reflects the biological variability of the maturation process and supports the value of a more individualized diagnostic framework.

Although the limited sample size precludes definitive conclusions, the consistency of certain trends observed in this analysis encourages further exploration. Rather than challenging the validity of Angelieri's method, our findings complement and refine its application by illustrating the potential diagnostic benefits of a multimodal CBCT approach—particularly in borderline cases—ultimately contributing to a more accurate and tailored treatment planning.

## Data Availability

The original contributions presented in the study are included in the article/Supplementary Material, further inquiries can be directed to the corresponding authors.
