# Supplementary material for: A new CBCT evaluation for individual assessment of midpalatal suture maturation: a retrospective analysis
Source: Front Oral Health. 2026 Jan 16;6:1630883. doi: 10.3389/froh.2025.1630883 (PMC12855418; doi:10.3389/froh.2025.1630883)
Supplement: Supplementary file 1 [file Table1.docx]

**Supplementary Table 1.** Description of the whole sample according to type of direction of difference between Angelieri and TC evaluation

|  | **Negative (one TC)** | **Positive**  **(one TC)** | **Positive**  **(>=2TC)** | **Complete**  **Concordance** | **p*** |
| --- | --- | --- | --- | --- | --- |
| **A** | 0 (0.00) | 0 (0.00) | 0 (0.00) | 1 (4.30) | 0.25 |
| **B** | 0 (0.00) | 1 (11.10) | 0 (0.00) | 1 (4.30) |  |
| **C** | 0 (0.00) | 7 (77.80) | 1 (100.00) | 9 (39.10) |  |
| **D** | 1 (100.00) | 1 (11.10) | 0 (0.00) | 5 (21.70) |  |
| **E** | 0 (0.00) | 0 (0.00) | 0 (0.00) | 7 (30.40) |  |

**Fisher’s exact test*

**Supplementary Table 2.** Description of the whole sample. N:34.

|  | Mean ± sd | Median (iqr) |
| --- | --- | --- |
| Age (years) | 18.30 ± 11.20 | 13.80 (7.43) |
| Sex |  |  |
| Female | 25 (73.50) |  |
| Male | 9 (26.50) |  |
| Agelieri Staging |  |  |
| A | 1 (2.90) |  |
| B | 2 (5.90) |  |
| C | 17 (50.00) |  |
| D | 7 (20.60) |  |
| E | 7 (20.60) |  |
| Corcordance | 2.65 ± 0.55 |  |
| Difference |  |  |
| Decreased ossification | 1 (2.90) |  |
| Increased ossification | 9 (26.50) |  |
| Incr./Incr. | 1 (2.90) |  |
| X | 23 (67.60) |  |
| Age Group |  |  |
| 5-<11 y | 4 (11.80) |  |
| 12-<14 y | 14 (41.20) |  |
| 14-18 y | 6 (17.60) |  |
| >18 y | 10 (29.40) |  |
